# Comparative Transcriptomic Analyses Reveal Differences in the Responses of Diploid and Triploid Eastern Oysters to Environmental Stress

**DOI:** 10.1111/eva.70028

**Published:** 2024-10-22

**Authors:** Rujuta V. Vaidya, Sarah Bodenstein, Dildorakhon Rasulova, Jerome F. La Peyre, Morgan W. Kelly

**Affiliations:** ^1^ Department of Biological Sciences Louisiana State University Baton Rouge Louisiana USA; ^2^ Louisiana Sea Grant College Program Louisiana State University Baton Rouge Louisiana USA; ^3^ School of Animal Sciences Louisiana State University Agricultural Center Baton Rouge Louisiana USA

**Keywords:** *Crassostrea virginica*, gene expression, Gulf of Mexico, mortality, ploidy, salinity

## Abstract

Triploid oysters are commonly used as the basis for production in the aquaculture of eastern oysters along the USA East and Gulf of Mexico coasts. While they are valued for their rapid growth, incidents of triploid mortality during summer months have been well documented in eastern oysters, especially at low salinity sites. We compared global transcriptomic responses of diploid and triploid oysters bred from the same three maternal source populations at two different hatcheries and outplanted to a high (annual mean salinity = 19.4 ± 6.7) and low (annual mean salinity = 9.3 ± 5.0) salinity site. Oysters were sampled for gene expression at the onset of a mortality event in the summer of 2021 to identify triploid‐specific gene expression patterns associated with low salinity sites, which ultimately experienced greater triploid mortality. We also examined chromosome‐specific gene expression to test for instances of aneuploidy in experimental triploid oyster lines, another possible contributor to elevated mortality in triploids. We observed a strong effect of hatchery conditions (cohort) on triploid‐specific mortality (field data) and a strong interactive effect of hatchery, ploidy, and outplant site on gene expression. At the low salinity site where triploid oysters experienced high mortality, we observed downregulation of transcripts related to calcium signaling, ciliary activity, and cell cycle checkpoints in triploids relative to diploids. These transcripts suggest dampening of the salinity stress response and problems during cell division as key cellular processes associated with elevated mortality risk in triploid oysters. No instances of aneuploidy were detected in our triploid oyster lines. Our results suggest that triploid oysters may be fundamentally less tolerant of rapid decreases in salinity, indicating that oyster farmers may need to limit the use of triploid oysters to sites with more stable salinity conditions.

## 1 Introduction

Polyploidy is the heritable condition in which a typically diploid cell or organism inherits an additional set (or sets) of chromosomes (de Sousa et al. 2016). Instances of polyploidy occur when chromosomes fail to separate normally during cell division, resulting in gametes that have an unequal distribution of chromosomes. Having extra sets of chromosomes affects many structural and physiological aspects of the cell—polyploid individuals are generally bigger in size, have faster growth rates, and offer better yields in commercially valuable species (Piferrer et al. 2009). The prospect of a bigger marketable product has generated great interest in the generation of polyploids in agriculture and aquaculture, including in commercially valuable bivalves like the eastern oyster. Generation of triploid and tetraploid eastern oysters was first reported in 1981 (Stanley, Allen, and Hidu 1981), and in later years, several standardized protocols for hatchery production of triploid and tetraploid oysters have been published (Wadsworth, Wilson, and Walton 2019; Yang 2022, Hudson and Murrary 2014) and adapted by oyster farms as their primary basis for production. Compared to the natural diploid oysters, triploid oysters generally grow faster and have reduced gametogenesis as a consequence of their triploidy (Allen Jr. and Downing, 1986; Wadsworth, Wilson, and Walton 2019; Matt and Allen 2021). Unlike diploid oysters, who invest considerable energy reserves in reproduction, triploid oysters instead channel that energy into their growth, resulting in better meat quality during summer (Meyers et al. 1991; Degremont et al. 2012; Walton et al. 2013). All of these “triploid advantages” have made them a desirable alternative to natural diploid oysters, and triploids have been adopted by many hatcheries as their primary basis for production (Brianik and Allam 2023).

However, in recent years there have been reports of triploid eastern oysters experiencing higher mortality than diploid oysters during the spring and summer months. This phenomenon, collectively termed “triploid mortality” has been observed along the USA mid‐Atlantic and Gulf of Mexico (GoM) coasts (George et al. 2023; Houssin et al. 2019; Matt et al. 2020; Wadsworth, Wilson, and Walton 2019). Several factors, such as unfavorable environmental conditions (salinity and temperature), maladapted parental stocks, and disrupted gene regulation and stress responses in triploid oysters, have been suggested to contribute to triploid mortality, however, the exact underlying cellular mechanisms causing these triploid mortality events remain unresolved (Auger et al. 2005; Callam, Allen, and Frank‐Lawale 2016; Ching et al. 2010; Hand, Nell, and Thompson 2004; Marshall, Coxe, et al. 2021; Marshall, Casas, et al. 2021; Swam et al. 2022). Additionally, chromosome losses are more frequent in polyploid organisms (Comai 2005), and instances of aneuploidy have been reported in early cell divisions of polyploid oysters (de Sousa et al. 2016; Wang et al. 1999). Losing a part of or a whole chromosome can cause major disruptions in patterns of gene expression and regulation, which can affect the susceptibility of triploid oysters to unfavorable environmental conditions.

In our study, we compared transcriptomic responses of diploid and triploid oysters bred from the same three maternal lines and outplanted to a high (annual mean salinity = 19.4 ± 6.7 psu) and low (annual mean salinity = 9.3 ± 5.0 psu) salinity site at the onset of the mortality event in the summer of 2021 to test for the effect of parental contribution on triploid performance. To investigate whether triploid oysters show disrupted gene regulation and stress responses, we compared the global transcriptomic response of diploid and triploid oysters to high and low salinity sites. We also tested for evidence of aneuploidy in triploid oysters used in the experiment by applying methods described in Griffiths, Scialdone, and Marioni (2017) to our transcriptomic dataset.

The oysters used in this study were part of a larger experiment measuring growth and physiology of triploid oysters at these sites; environmental data (salinity and temperature) and morphological measurements including growth, *Perkinsus marinus* (dermo) infection prevalence, and mortality are reported in Bodenstein, Casas, Tiersch and La Peyre (2023), Bodenstein et al. (2023). Interestingly, Bodenstein, Casas, Tiersch and La Peyre (2023), Bodenstein et al. (2023) found no effect of maternal stocks on triploid survival but found a strong effect of hatchery conditions (referred to as cohorts), with one of the two hatcheries used for rearing oyster lines showing higher triploid mortality. We therefore decided to test for effects of hatchery, ploidy, and site on gene expression to identify gene expression patterns associated with elevated mortality risk in triploid oysters during summer conditions.

## 2 Methods

### 2.1 Oyster Crossing and Outplanting

**FIGURE 1:**
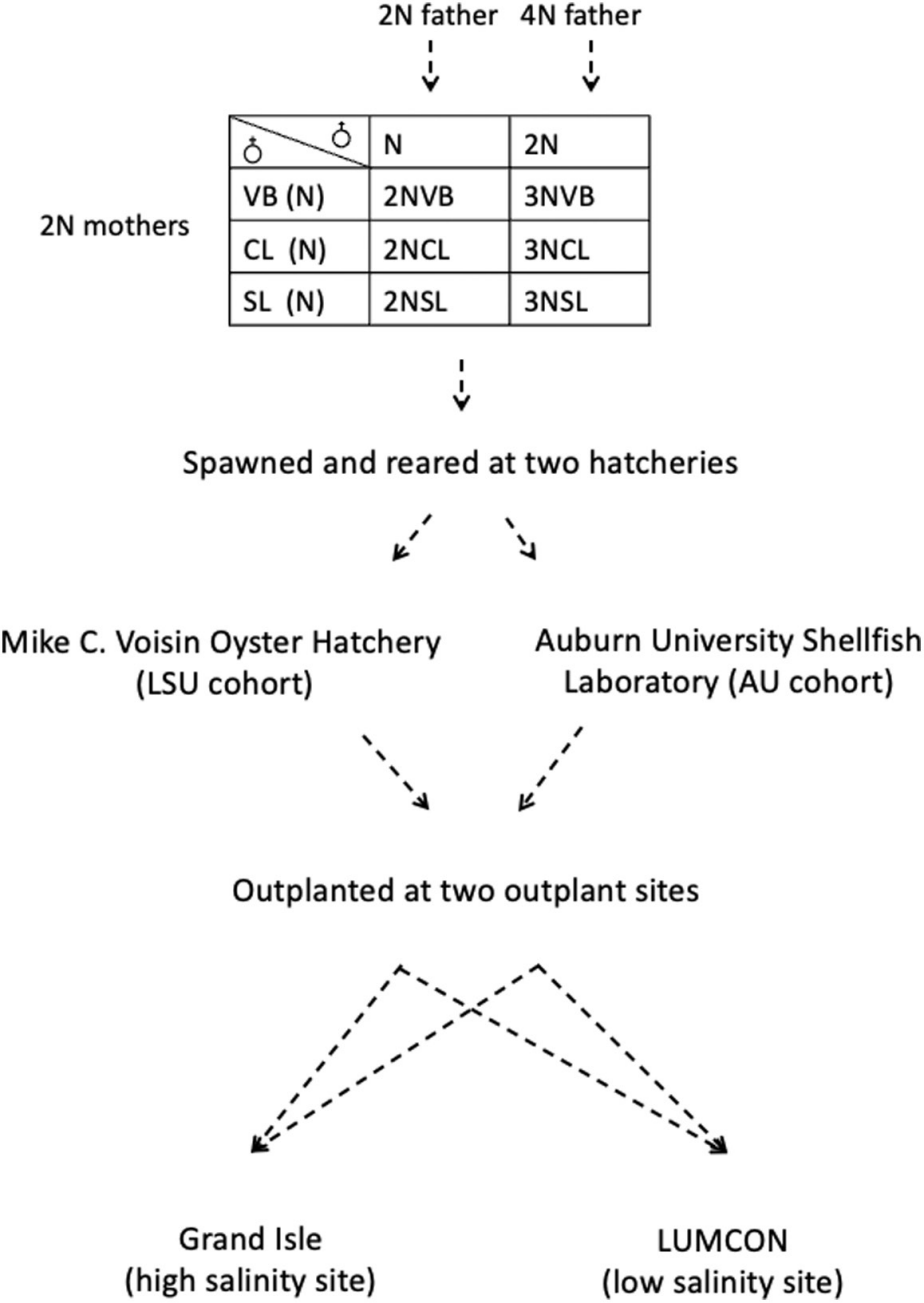
Cross strategy used to generate diploid and triploid lines for this study. Detailed cross strategy and number of oysters used for each cross are described in Bodenstein, Casas, Tiersch and La Peyre (2023); Bodenstein et al. (2023).

The detailed cross strategy and outplanting designs are explained in Bodenstein, Casas, Tiersch and La Peyre (2023), Bodenstein et al. (2023) and summarized in Figure 1. Briefly, to establish diploid parental broodstocks adapted to different salinity regimes, oysters were collected from each of three Louisiana public oyster grounds that had different salinity regimes: Calcasieu Lake (CL, annual mean salinity = 16.2 ± 2.8 [± standard deviation (SD), *n* = 10, 2009–2018]), Sister Lake (SL, annual mean salinity = 11.2 ± 5.5), and Vermilion Bay (VB, annual mean salinity = 7.4 ± 1.6). The tetraploids used in this study were LSU 4DGNL17 (maintained at the Louisiana Sea Grant Oyster Research Farm) and AU 4MC18 (maintained at the Grand Bay Oyster Park, Alabama). Oysters were conditioned and spawned in June 2019 at Mike C. Voisin Oyster Hatchery (LSU cohort) and at the Auburn University Shellfish Laboratory (AU cohort) in Dauphin Island, AL, in July 2019 as described in Bodenstein, Casas, Tiersch and La Peyre (2023), Bodenstein et al. (2023). Depending on where they were spawned, F1 progeny were sorted into LSU (Louisiana State University) or AU (Auburn) cohorts. A total of six crosses were produced at each hatchery. The diploid F1 crosses were produced by crossing male and female oysters from each wild broodstock population (CL, SL, and VB), and the triploid F1 crosses were produced by crossing females from wild broodstock populations with males from two tetraploid lines (one from each hatchery, Figure 1). Diploid and triploid crosses were generated from the same females at the Auburn hatchery and were therefore half siblings. However, at the LSU hatchery, diploid and triploid crosses were not half siblings and, in certain cases, were produced on separate days. Oysters were reared and transported to their final outplant sites as described in Bodenstein, Casas, Tiersch and La Peyre (2023), Bodenstein et al. (2023). Oysters from both cohorts were outplanted to a low (near the Louisiana Universities Marine Consortium [LUMCON], annual mean salinity = 9.3 ± 5.0) and a moderate salinity site (Grand Isle, annual mean salinity = 19.4 ± 6.7) in Louisiana. Oysters from the Auburn cohort were outplanted in November 2019 at the two sites, and oysters from the LSU cohort were outplanted at Grand Isle in December 2019 and at LUMCON in January 2020. At each site, four replicate baskets containing 80 oysters each were outplanted for each of the six crosses of each ploidy in the two cohorts, for a total of 48 baskets per site. Growth and mortality measurements were performed monthly until November 2020 and are described in Bodenstein, Casas, Tiersch and La Peyre (2023), Bodenstein et al. (2023).

### 2.2 RNA Sequencing

On 8^th^ July, 2020, eight individuals per cross were sampled at both outplant sites. Approximately 0.5‐cm^2^ piece of gill tissue was dissected in the field from each individual and preserved with Invitrogen RNAlater. Total RNA was extracted using an E.Z.N.A. Total RNA Kit I (Omega BIO‐TEK; VWR catalog no. 101319) following the manufacturer's instructions. The yield and quantity were assessed using a NanoDrop 2000 spectrophotometer. Total RNA extracted from the 192 individuals was sent to the University of Texas at Austin's Genomic Sequencing and Analysis Facility, where RNA quality control was confirmed using a 2100 Agilent Bioanalyzer on an Eukaryote Total RNA Nano chip and libraries were produced using the Tag‐sequencing approach (Meyer, Aglyamova, and Matz 2011). The resulting 192 libraries were sequenced equally across two lanes of an Illumina HiSeq 2500 platform, with 100‐bp single‐end reads. Adapter sequences were trimmed using trimmomatic (version 0.39) (Bolger, Lohse, and Usadel 2014), and base pairs with quality scores below 35 were removed. The trimmed reads were then mapped to the *C. virginica* reference genome (Gómez‐Chiarri et al. 2015) with known haplotigs removed (https://github.com/jpuritz/OysterGenomeProject/tree/master/Haplotig_Masked_Genome) using star rna‐seq aligner (single pass option). Reads were mapped to gene features with the options (−quantMode GeneCounts–outFilterScoreMinOverLread 0.50–outFilterMatchNminOverLread 0.50) to adjust for poly‐A tail contamination (Sirovy et al. 2021) to finally generate a count matrix using ReadsPerGene.out.tab output.

### 2.3 Differential Gene Expression

Differential gene expressions (DEGs) were calculated using DESeq2 (v 1.24.0) (Love, Huber, and Anders 2014), with a false discovery rate (FDR)‐adjusted *p* value cutoff of 0.01. Since mortality data reported in Bodenstein, Casas, Tiersch and La Peyre (2023), Bodenstein et al. (2023) showed that maternal parentage did not have any effect on triploid mortality, we did not include dams in our pairwise DEG comparisons. The mortality data showed a strong effect of cohort on triploid survival, and we therefore incorporated cohorts in our DEG comparisons. We performed pairwise comparisons of diploid and triploid oysters across cohorts and ploidy. Gene ontology enrichment was also performed using GO_MWU (Wright et al. 2015), using the Fisher's Exact Test (*p* < 0.05) and the scripts available at: https://github.com/z0on/GO_MWU/blob/master/GO_MWU.R.

### 2.4 Weighted Gene Correlation Network Analysis

The R package weighted gene correlation network analysis (WGCNA) (Langfelder and Horvath 2008) was used to identify clusters of genes with highly correlated expression patterns. We filtered out genes with fewer than five counts per million in 80% of all samples to remove lowly expressed features, retaining only samples with greater than five counts per million in 80% of all samples. The WGCNA was run using 9172 genes that passed this criterion, using a soft threshold value of 12, a minimum module size of 30, and a signed adjacency matrix. The network was then correlated to site, cohort, and ploidy, with variables converted to binary format. Module eigengenes, defined as the first principal component of a given module (Langfelder and Horvath 2008), were visualized using ggplot2, and GO enrichment analysis was conducted using GO_MWU. To measure the magnitude of the difference between transcriptomic responses of our diploid and triploid oysters, we weighted the samples by the percent of variation explained by each axis and then calculated the shift (Euclidean distances) in multivariate space between diploid and triploid samples matched for their cohort, site, and dams.

### 2.5 Testing for Aneuploidy in Triploid Oysters

We also assessed for instances of aneuploidy across all 10 oyster chromosomes. To do this, we first categorized our samples into diploid and triploid pairs by matching their site, cohort, and dam; that is, a diploid oyster from the Auburn cohort and VB dam broodstock that was outplanted to Grand Isle was paired with a triploid oyster from the Auburn cohort and VB dam broodstock and was outplanted to Grand Isle. For every pair, we then plotted the normalized log transformed counts per millions (CPM) values such that each point on the plot represented a transcript and *X* and *Y* coordinates of every point were CPM values of that transcript in the diploid and triploid oyster, respectively. For each pair compared, ten such plots (one for each chromosome) were made, and the slope of the regression line was calculated. As the CPM values were normalized to the library sizes, change in the slope for any chromosome for a particular sample pair was considered as an indicator of aneuploidy occurring at that chromosome: while differential gene expression should result in differences in transcript levels between diploids and triploids for individual genes, only aneuploidy is expected to lead to chromosome‐wide differences in transcript levels between individuals for individual chromosomes after normalizing to library size (Griffiths, Scialdone, and Marioni 2017; Stamoulis et al. 2019; Stingele et al. 2012; Zhao et al. 2017).

## 3 Results

### 3.1 Principal Component Analysis and Aneuploidy Analysis

**FIGURE 2:**
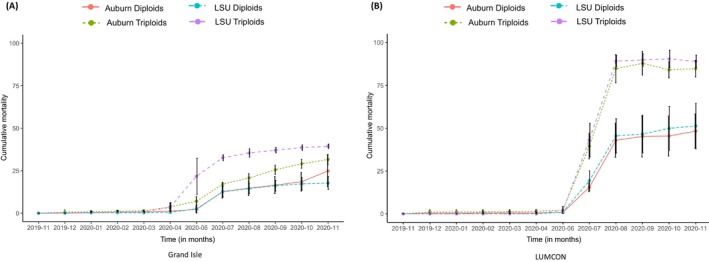
Cumulative mortality of diploid and triploid oysters outplanted to (A) Grand Isle and (B) LUMCON sites, replotted from Bodenstein, Casas, Tiersch and La Peyre (2023), Bodenstein et al. (2023). The *X*‐axis indicates timepoints in months for which mortality data was collected, and the *Y*‐axis represents cumulative mortality. Oysters are grouped together based on their ploidy and cohorts. Purple line: Triploid oysters from LSU cohort; green dotted line: Triploid oysters from Auburn cohort; blue dotted line: Diploid oysters from LSU cohort; orange line: Diploid oysters from Auburn the cohort.

**FIGURE 3:**
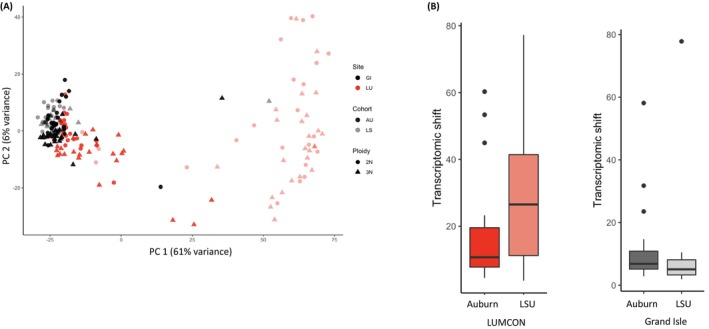
(A) PCA showing separation based on site, cohort, and ploidy; (B) transcriptomic shift measured as distance in multivariate space between diploid and triploid samples matched for their site, cohort, and dams.

Detailed mortality and growth measurements are described in Bodenstein, Casas, Tiersch and La Peyre (2023), Bodenstein et al. (2023). Overall, triploids showed higher mortality at the low salinity site (LUMCON) (Figure 2). At both high and low salinity sites, the cumulative mortality of triploids was higher than diploids, and triploids from the LSU cohort experienced higher cumulative mortality than the AU cohort. Maternal stock (dams) did not influence the cumulative mortality of triploids at either site. To visualize broad patterns of gene expression, a principal component analysis (PCA) was performed. Raw count data for diploid and triploid oysters was first normalized using the variance stabilizing transformation (VST) function in the DESeq2 package, and a PCA was performed using the plotPCA function in the DESeq2 package. The first two principal component axes accounted for 61% and 6% variance in samples, respectively (Figure 3A). Out of the four variables, outplant site had the greatest effect on sample clustering, followed by cohort and ploidy. In line with the phenotypic data, we did not see any clustering based on dams. The magnitude of transcriptomic differences between diploid and triploid oysters at each site was visualized using PCA as per Thomas et al. (2022). Briefly, we extracted PC1 and PC2 values for each sample (represented by a point in the PCA plot) to calculate Euclidean distances between each diploid and triploid sample pair matched for their cohort, site, and dams. The distances were plotted as separate box plots for Grand Isle and LUMCON sites and color coded by their cohorts. Overall, the difference in magnitude was higher in diploid vs. triploid oyster comparisons at the LUMCON site, regardless of their cohort. To check if the differences in mortality were a result of aneuploidy in triploid oysters, we plotted normalized log transformed CPM values of diploid and triploid oysters on the *X* and *Y* axis respectively. Slope values for all ten chromosomes for each sample pair are summarized in Table S1. We did not see change in slopes of any chromosomes for any of our sample pairs, indicating no change in the triploid:diploid chromosome ratio across all ten chromosomes and thus no aneuploidy.

### 3.2 Differential Gene Expression Analysis

The results of our DEG analyses are summarized in Table 1. This analysis was divided into two major sections: across ploidy (diploid vs. triploid) and within ploidy (comparing oysters of the same ploidy across sites and cohorts). For the first section, we performed a total of four pairwise comparisons, each comparing diploid and triploid oysters belonging to a specific site + cohort combination. The pairwise comparison of diploid versus triploid oysters that were outplanted to a low salinity site (LUMCON) and belonged to a cohort associated with high mortality risk (LSU) yielded the highest number of DEGs (5522). Gene ontology (GO) analysis of these DEGs showed enrichment of GO terms involved in microtubules, cell cycle, and calcium binding. Specifically, triploid oysters from this comparison downregulated transcripts coding for genes related to calcium signaling (Calmodulin‐a, Histidine‐rich calcium binding protein (HRC), fibrillin1 & 2, annexin A4, cadherin‐23, and cadherin EGF LAG seven‐pass G‐type receptor 1[CELSR1]) and cell cycle processes (KIF2A, KIF3C, NEK4, NEK6, CDK1, TNKS1, HAUS augmin‐like complex subunit 3, MCM7, CHTF18, SMCs, RTEL1, and MAPKKK). On the other hand, a pairwise comparison of diploid versus triploid oysters that were outplanted to a high salinity site (Grand Isle) and belonged to a cohort associated with high mortality risk (LSU) yielded the lowest number of DEGs (1860). GO analysis for this comparison did not yield any GO terms. Notably, the pairwise comparison of diploid versus triploid oysters that were outplanted to high salinity sites (Grand Isle) and belonged to cohorts associated with low mortality risk (AU) revealed that the triploid oysters in this comparison upregulated transcripts that were enriched in GO terms related to reactive oxygen species and superoxide metabolic processes. Finally, our last pairwise comparison of diploid versus triploid oysters that were outplanted to the lower salinity site (LUMCON) and belonged to cohorts associated with low mortality risk (AU) yielded 2652 DEGs, and GO analysis for this comparison did not yield any GO terms.

**TABLE 1:** Number of DEGs detected for each pairwise comparison performed using DESeq2.

Comparison	DESeq2
2n LSU vs. 3n LSU at GI	1860
2n LSU vs. 3n LSU at LUMCON	5522
2n AU vs. 3n AU at GI	1925
2n AU vs. 3n AU at LUMCON	2652
2n AU vs. 2n LSU at LUMCON	10,180
2n AU vs. 2n LSU at GI	2232
3n AU vs. 3n LSU at LUMCON	11,810
3n AU vs. 3n LSU at GI	1371

For the second section, we performed within ploidy comparisons to examine if oysters of the same ploidy (i.e., either diploids or triploids) responded differently to outplant sites based on their cohorts. We performed four pairwise comparisons that were parallel to our first section, and the results are summarized in Table 1. Our pairwise comparison of triploid oysters belonging to cohort associated with low mortality risk (Auburn) versus cohort associated with high mortality risk (LSU) at low salinity site yielded higher (11810) DEGs than the same comparison performed for high salinity site (1371 DEGs). Similarly, our pairwise comparison of diploid oysters belonging to cohorts associated with low mortality risk (Auburn) versus cohorts associated with high mortality risk (LSU) at low salinity site also yielded higher (10180) DEGs than the same comparison performed for high salinity site (2232 DEGs). Gene ontology (GO) analysis for these DEGs revealed enrichment of many GO terms related to cell cycle and cell cycle regulation in transcripts upregulated by triploid oysters belonging to cohorts associated with high mortality risk (LSU) at low salinity outplant site (LUMCON). Downregulated transcripts from the same comparison revealed enrichment of GO terms related to ion transport, cell adhesion, and regulation of cell death. Similarly, GO analysis also revealed enrichment of GO terms related to cell cycle, DNA recombination, and DNA repair in transcripts upregulated by diploid oysters belonging to cohort associated with high mortality risk (LSU).

### 3.3 Weighted Gene Correlation Network Analysis

**FIGURE 4:**
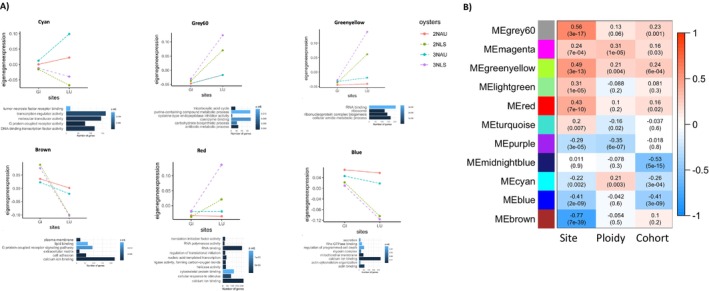
(A) Eigengene expression plots for four modules showing significant correlation to site and cohort in WGCNA, with samples grouped together by their cohorts and ploidies; and GO terms with the highest *p* values in those corresponding modules. Orange: Diploids from the Auburn cohort; green: Diploids from the LSU cohort; blue: Triploids from the Auburn cohort; purple: Triploids from the LSU cohort. (B) Heatmap of WGCNA module‐trait relationship results. The *X*‐axis shows the experimental parameters of interest used for correlations. Red indicates positive correlations, and blue indicates negative correlations between a module and an experimental parameter.

We also performed weighted gene correlation network analysis (WGCNA) to identify groups of genes displaying similar expression patterns in response to site, ploidy, and cohort. Figure 4B shows 11 modules labeled as color names and their degree of correlation with traits of interest. We found four (grey60, brown, red, and blue) modules associated with site and two (midnight blue and blue) with cohort. The module blue showed significant association with both site and cohort, while module cyan was associated with site, ploidy, and cohort. Figure 4B shows eigengene expression plots for brown, grey60, red, cyan, greenyellow, and blue modules with lines indicating samples grouped by their ploidies and cohorts.

Of interest are the expression patterns in the cyan module, where eigengene expression was higher for triploid oysters, but oysters from the Auburn cohort overall showed higher eigengene expression regardless of their ploidies. Similarly, eigengene expression in the red module was higher in oysters belonging to the LSU cohort but remained unchanged for oysters from the Auburn cohort. The red module was positively correlated to site, and GO terms overrepresented in this module were related to initiation and regulation of translation, calcium ion binding, cytoskeleton protein binding, and cellular response to stimulus. The blue module was negatively correlated with site and cohort and contained genes that were downregulated by oysters from the LUMCON site and LSU cohort. GO analysis for this module revealed an overrepresentation of GO terms related to calcium ion binding and actin binding. The brown module also showed strong negative correlation with site, that is, genes from this module were downregulated by oysters from the LUMCON site when compared with oysters from Grand Isle. GO analysis revealed enrichment of GO terms related to plasma membrane, lipid binding, cell adhesion, calcium binding, and extracellular matrix. On the other hand, Grey60 module was positively correlated to site, and GO analysis for this module revealed overrepresentation of GO terms related to TCA cycle and cysteine type endopeptidase inhibitor activity, among others. Eigengene plots from Figure 4A reveal eigengene expression patterns for the six modules described above. In particular, the eigengene expression pattern observed for diploid and triploid oysters in modules blue and brown is in agreement with our DEG results, that is, the magnitude of eigengene expression observed for triploids in response low salinity site is lower than diploids in these two modules. On the other hand, modules grey60, greenyellow, and red revealed additional ploidy and cohort‐specific expression patterns that were not detected in our DEG analysis. Eigengene expression pattern in module grey60 reveals a cluster of genes that are upregulated in response to low salinity site; however, the magnitude of eigengene expression is identical for diploids and triploids in cohort associated with low mortality risk (Auburn). Additionally, the magnitude of eigengene expression observed for triploids in this module is higher than that of diploids in cohort associated with high mortality risk (LSU). Taken together, module grey60 highlights a unique cluster of genes that is upregulated exclusively by triploid oysters of cohort associated with high mortality risk (LSU) in response to the low salinity outplant site. GO analysis performed on these genes revealed enrichment of GO terms related to tricarboxylic acid cycle, coenzyme binding, and carbohydrate biosynthetic processes.

Similarly, modules greenyellow and red highlight another unique cluster of genes that are upregulated in response to low salinity in diploid and triploid oysters belonging to the cohort associated with high mortality risk (LSU), but not in oysters belonging to the cohort associated with lower mortality risk (Auburn). GO analysis performed on these genes revealed enrichment of GO terms related to RNA binding, ribosome, cellular response to stimulus, RNA polymerase activity, and helicase activity.

## 4 Discussion

The primary aim of our study was to examine whether hatchery conditions, ploidy, and outplant site influence transcriptomic responses in triploid oysters and whether particular patterns of expression are associated with elevated mortality risk during summer conditions. Mortality data revealed no effect of maternal broodstocks on triploid mortality. Our analyses also did not detect instances of aneuploidy, which has been previously observed in polyploid organisms (Comai 2005). On the other hand, mortality data from Bodenstein, Casas, Tiersch and La Peyre (2023), Bodenstein et al. (2023) showed a strong effect of cohort, which was also reflected in our transcriptomic analysis. Our comparisons of diploid and triploid oysters across outplant sites and cohorts revealed a significant number of DEGs and GO terms related to key biological processes such as calcium signaling and cell cycle processes, indicating the stronger influence of these two factors on survival under stressful summer conditions. Our inter‐ and intraploidy analyses reveal that diploid and triploid oysters share at least some of their cellular response to low salinity and summer conditions, as seen through enrichment of common GO terms and transcripts.

### 4.1 Intra‐Ploidy Variation in Transcriptomic Response to Salinity/Outplant Sites

Our within‐ploidy DEG comparisons, performed separately for diploid and triploid oysters, revealed that cell division was significantly affected in both ploidies at the low salinity site. At the low salinity site, both ploidies from the higher mortality LSU cohort upregulate transcripts that were enriched for GO terms related to DNA replication and repair, cell cycle regulation, and response to stimulus. The presence of these common GO terms indicates that regardless of their ploidies, both diploid and triploid oysters respond to low salinity cues by altering or arresting their cell division. Upregulation of genes involved in cell cycle checkpoints in the gill tissue has been previously reported in mussels (Lockwood and Somero 2011) and killifish (Kültz and Avila 2001; Whitehead et al. 2011), indicating that cell cycle regulation is a conserved response to low salinity across species. Changes in salinity have a multifaceted impact on a cell—they can change the osmotic balance inside the cell, activate the cellular signaling pathways that detect changes in salinity and affect the structure and functions of proteins and nucleic acids in the cell. Oysters are osmoconformers; however, adjusting to abrupt and large changes in salinity, can take days and is a function of the initial salinity and the rate, magnitude, and direction of the salinity change (Shumway, 1977; Hand and Stickle 1977; La Peyre et al. 2003; La Peyre, Gossman, and La Peyre 2009; McFarland, Donaghy, and Volety 2013). Therefore, response to salinity stress has been proposed to be a complex process that targets multiple functions, including the initiation of a damage control response to repair the protein and DNA damage caused by salinity change and also initiating a response to remove molecules or cells that have been damaged beyond repair (Hochachka and Somero, 2002). The enrichment of DNA repair GO terms we see for the LSU cohort therefore suggests that they were employing similar stress response mechanisms to combat low salinity stress. Arresting growth during salinity stress is a way of ensuring that damaged DNA isn't passed down in the cell division cycles—it gives the cell enough time to repair the DNA damage and then progress to cell division. The upregulation of cell cycle checkpoints, which are essentially “quality checkpoints” points to ensure the structural integrity of the DNA before progressing to the next step, is also an indicator of LSU triploids coping with low salinity stress.

This shared response across ploidies presents an interesting case, as in our next set of comparisons, we observe that, when compared to each other, the magnitude of upregulation for genes involved in the salinity response is lower in triploids than diploids. The shared transcriptomic response to salinity and its decreased magnitude in triploids could mean that triploids mount a weaker adaptive response to salinity, resulting in greater cellular damage. This pattern, where less stress‐tolerant genotypes mount a weaker adaptive transcriptomic response to a stressor, is a phenomenon that has been observed in other transcriptomic studies seeking to understand physiological mechanisms underlying intra‐specific variation in stress tolerance (DeBiasse and Kelly 2016).

Along with these shared patterns, we also observed some ploidy‐specific gene expression patterns for triploid oysters. At the lower salinity site, triploid oysters from the higher mortality cohort upregulated transcripts involved in immune response compared to their low‐mortality counterparts. In bivalves, abiotic stressors such as temperature and salinity have been shown to induce transcriptional changes related to immune response (Ellis et al. 2011; Gagnaire et al. 2006; Lacoste et al. 2002; Place, Menge, and Hofmann 2012). The upregulation we observed in the triploid oysters at the low salinity site could potentially be due to the stressful salinity and temperature conditions they experienced.

### 4.2 Interploidy Variation in Transcriptomic Response to Salinity/Outplant Sites

At the low salinity site, triploid oysters from the high mortality cohort downregulated genes involved in three key processes—calcium signaling, ciliary activity, and cell cycle checkpoints. The GO terms and transcripts enriched for these processes hint at some degree of crosstalk among these three processes, suggesting a coordinated stress response in response to salinity, led by calcium signaling. Intracellular calcium is noted for its role as a messenger molecule that facilitates intra‐ and intercellular communication. Specifically, an increase in intracellular calcium in response to abiotic stress is a hallmark of plant stress response, and a similar increase in calcium and its receptors (partners) in response to ocean acidification has been noted in the Pacific oyster as well (Xin et al. 2022). Unlike their diploid counterparts, triploid oysters were unable to upregulate genes involved in calcium signaling, indicating triploid‐specific problems in detecting and responding to low salinity stress. In our study, triploid oysters from the high mortality cohort downregulated several key members of the calcium‐mediated stress response pathway, such as Calmodulin‐a, Histidine‐rich calcium‐binding protein (HRC), and cadherin EGF LAG seven‐pass G‐type receptor 1(CELSR1). Calmodulin‐a is a well‐studied calcium‐binding protein that influences key physiological processes like cell proliferation, differentiation, and apoptosis by interacting with its many downstream targets (Xin et al. 2022). Histidine‐rich calcium binding protein (HRC), which is important for calcium homeostasis, and cadherin EGF LAG seven‐pass G‐type receptor 1(CELSR1), a type of G protein‐coupled transmembrane receptor instrumental for communication between extracellular and intracellular environments, were also downregulated by triploid oysters from the high mortality cohort at the low salinity site (Arvanitis et al. 2011; Wang et al. 2014). Furthermore, genes encoding calcium‐dependent proteins responsible for structural elements of intracellular and extracellular space like fibrillin1 & 2, annexin A4, and cadherin‐23 were also downregulated by triploid oysters from the high mortality cohort at the low salinity site. Changes in cell shape and volume do occur in response to salinity, and downregulation of these cytoskeleton elements by triploid oysters may have hindered their effort to maintain their cell shape and osmotic balance under low salinity conditions (Zhang et al. 2017; Hochachka and Somero, 2002). Numerous genes coding for cytoplasmic and axonemal dyneins were also downregulated by triploid oysters under stress. As one of the three major cytoskeletal motor proteins, cytoplasmic and axonemal dyneins have been shown to be associated with sensory and motility functions of the cilia, respectively. Upregulation of transcripts involved in ciliary activity at low salinity has been observed in diploid *C. virginica* and has been hypothesized to help in maintaining homeostasis and avoiding valve closure (Jones, Johnson, and Kelly 2019). The lack of upregulation of these genes in triploid oysters, combined with the heightened risk of mortality at the low salinity site, could be an indication of differences in ciliary activity in their gill tissue, and future experiments measuring ciliary activity in triploid oysters under summer stress would be helpful to understand the role it may play in triploid mortality. Along with ciliary activity, triploid oysters from the LSU cohort also downregulated genes involved in cell cycle and its regulation, such as KIF2A, KIF3C, NEK4, NEK6, CDK1, TNKS1, HAUS augmin‐like complex subunit 3, and MAPKKK, in response to low salinity.

This downregulation of several key players involved in cell cycle processes could be an indication of gill tissue cells of triploid oysters encountering errors at multiple stages of cell division. As mentioned in the section about intraploidy comparisons, upregulation of genes involved in cell cycle was a shared transcriptomic response observed in both ploidies, but its magnitude appears to be lower in triploid oysters when compared to diploids. This ploidy‐specific difference in gene expression could be due to the complicated nature of cell division in polyploid individuals. Cell division in polyploid cells, especially those with odd numbers of chromosomes such as triploids, has been shown to be prone to errors such as chromosome loss and aneuploid cells (Comai 2005). Additionally, progression through the cell cycle does depend on environmental stimuli like thermal and osmotic stress (Hochachka and Somero, 2002). It is possible that the triploid oysters at low salinity sites were particularly challenged with the dual threat of unfavorable environmental conditions and an unfavorable cellular milieu for optimal cell division.

The cohort effect in our interploidy comparisons could be attributed to differences in the two hatchery conditions. At the low salinity site, field mortality measurements from Bodenstein, Casas, Tiersch and La Peyre (2023), Bodenstein et al. (2023) show higher cumulative mortalities for triploids from the LSU cohort but do not see a cohort‐specific effect on diploid mortality. The cohort effect was also observed in growth rates—at low salinity sites, both diploids and triploids from the LSU cohort had slower growth rates. Since the point of distinction between two cohorts was hatchery conditions, we think that early life experience could be influencing growth and survival in later life. Stressors present in the early life environment of oysters have been shown to have an impact on their later life survival (Donelan, Breitburg, and Ogburn 2021; Hettinger et al. 2012; Spencer et al. 2020), and the cohort‐specific differences in growth rates and survival observed in this experiment could be due to different rearing conditions experienced by oyster larvae at the two hatcheries.

## 5 Conclusion

Use of triploid individuals in aquaculture has become a popular aquaculture technique, especially for commercially important species of bivalves and fish (Nell 2002; Xiang et al. 2006; Budiño et al. 2006; Fraser et al. 2012). However, triploid individuals have also been shown to be more susceptible to environmental stressors such as changes in temperature, salinity, and oxygen availability (Ojolick et al. 1995; Hansen et al. 2015; Meyers et al. 1991; Bodenstein, Casas, Tiersch and La Peyre 2023; Bodenstein et al. 2023). Our data indicate problems with cell cycle checkpoints and a dampened transcriptomic stress response for triploid oysters at the low salinity site. This suggests that triploid oysters may be fundamentally less tolerant of environmental stress, especially rapid changes in salinity. As a result, oyster farmers may need to limit the use of triploid oysters to sites with more stable environmental conditions.

## Conflicts of Interest

The authors declare no conflicts of interest.

## Supporting information


**Data S1:** Enriched GO terms detected in differential gene expression analysis and WGCNA.

**Data S2:** R script used for PCA and DEG analysis.

**Data S3:** R script used for calculating transcriptomic shift.

**Data S4:** R script used for WGCNA analysis.

**Table S1:** Regression line slopes calculated for ten chromosomes for each diploid and triploid oyster pair, matched for their site, cohort, and dam.

**Figure S1:** PCAs showing separation based on (A) Site, (B) Cohort, (C) Ploidy, and (D) Dams.

Data S5.

Data S6.

## Data Availability

The raw data files used in the study are available on NCBI’s SRA. The accession number is PRJNA1171449.
