# Supplementary figures and images for: Comparative Transcriptomic Analyses Reveal Differences in the Responses of Diploid and Triploid Eastern Oysters to Environmental Stress
Source: Evol Appl. 2024 Oct 22;17(10):e70028. doi: 10.1111/eva.70028 (PMC11496204; doi:10.1111/eva.70028)

## Slide 1
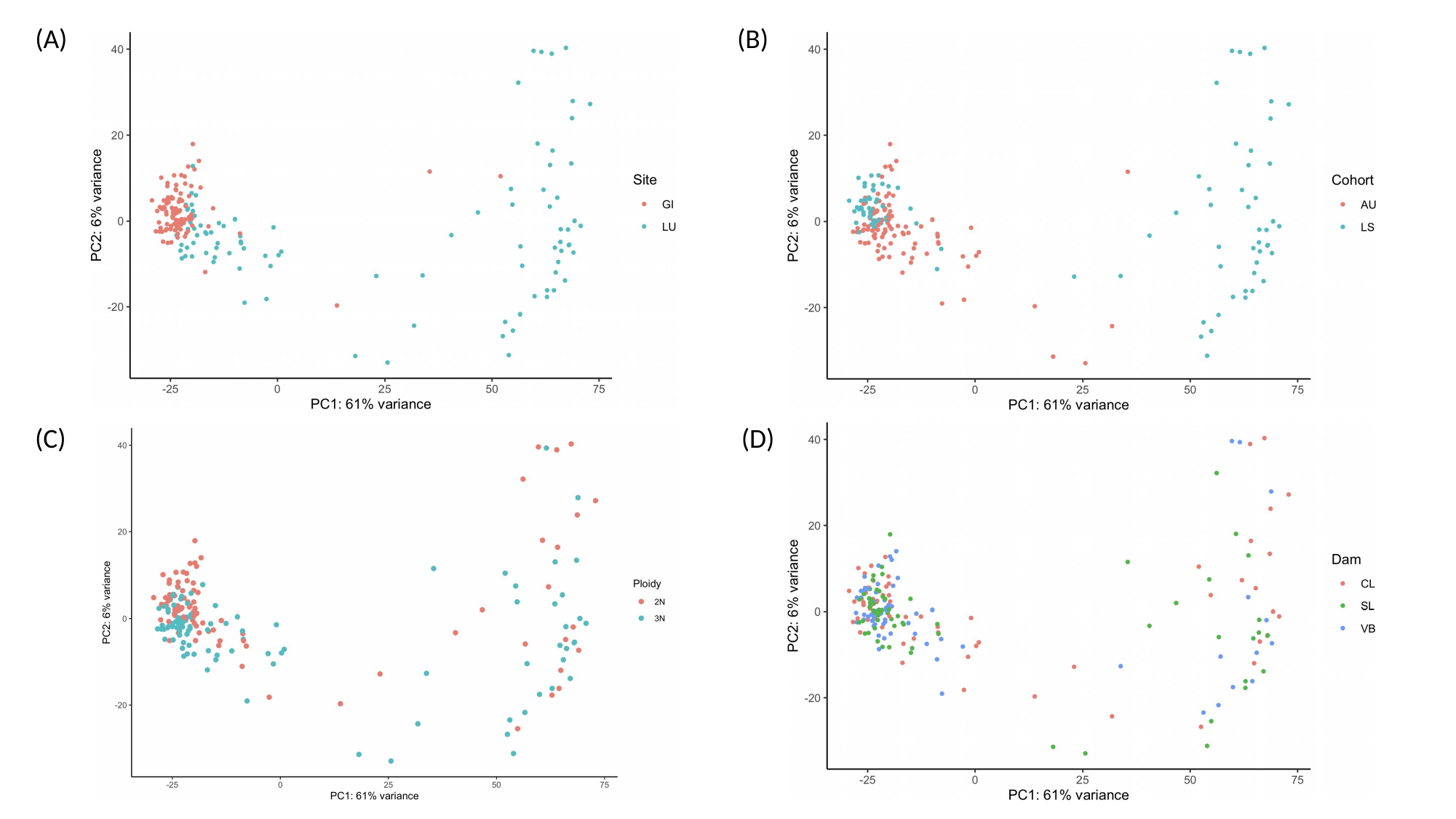

(A)
(B)
(C)
(D)

Supplement: Supplementary file 6 — Figure S1: PCAs showing separation based on (A) Site, (B) Cohort, (C) Ploidy, and (D) Dams. [file EVA-17-e70028-s007.pptx]
