# Supplementary material for: Comparative Transcriptomic Analyses Reveal Differences in the Responses of Diploid and Triploid Eastern Oysters to Environmental Stress
Source: Evol Appl. 2024 Oct 22;17(10):e70028. doi: 10.1111/eva.70028 (PMC11496204; doi:10.1111/eva.70028)

Supplementary table 1: Regression line slopes calculated for ten chromosomes for each diploid and triploid oyster pair, matched for their site, cohort, and dam.

GO terms obtained for DESeq2 comparisons of diploid and triploid oysters

Supplementary table 2: Diploid vs triploid oysters from LSU cohort at LUMCON (downregulated DEGs)

Supplementary table 3: Diploid vs triploid oysters from Auburn cohort at Grand Isle (upregulated DEGs).

Supplementary table 4: Auburn diploid oysters vs LSU diploid oysters at LUMCON (upregulated DEGs).

Supplementary table 5: Auburn triploid oysters vs LSU triploid oysters at LUMCON (upregulated DEGs)

GO terms for WGCNA modules:

Supplementary table 6: Red module

| **Composition** | **pval** | **level** | **nseqs** | **term** | **name** | **GO_Category** |
| --- | --- | --- | --- | --- | --- | --- |
| 12/19 nuclear transcription factor complex | 0.0148679 |  |  |  | nuclear transcription factor complex | Cellular Component |
| 9/15 replication fork | 0.06315854 | 3 | 15 | GO:0005657 | replication fork | Cellular Component |
| 38/90 chromosomal part | 0.01750801 | 2 | 90 | GO:0044427 | chromosomal part | Cellular Component |
| 6/8 Ino80 complex | 0.05740362 | 5 | 8 | GO:0031011;GO:0033202 | Ino80 complex | Cellular Component |
| 9/11 INO80-type complex | 0.00455291 | 3 | 11 | GO:0097346 | INO80-type complex | Cellular Component |
| 49/112 transferase complex | 0.00255289 | 2 | 112 | GO:1990234 | transferase complex | Cellular Component |
| 5/6 nuclear DNA-directed RNA polymerase complex | 0.05740362 | 2 | 6 | GO:0055029;GO:0000428 | nuclear DNA-directed RNA polymerase complex | Cellular Component |
| 14/29 transferase complex, transferring phosphorus-containing groups | 0.09045203 | 2 | 29 | GO:0061695 | transferase complex, transferring phosphorus-containing groups | Cellular Component |
| 53/98 nucleoplasm part | 6.01E-07 | 2 | 98 | GO:0044451 | nucleoplasm part | Cellular Component |
| 17/25 acetyltransferase complex | 0.00068229 | 3 | 25 | GO:0000123;GO:0031248;GO:1902493 | acetyltransferase complex | Cellular Component |
| 9/12 H4 histone acetyltransferase complex | 0.01111106 | 8 | 12 | GO:0035267;GO:0043189;GO:1902562 | H4 histone acetyltransferase complex | Cellular Component |
| 12/22 mitochondrion | 0.05740362 | 2 | 22 | GO:0005739 | mitochondrion | Cellular Component |
| 23/27 nucleolus | 4.08E-08 | 3 | 27 | GO:0005730 | nucleolus | Cellular Component |
| 79/192 intracellular non-membrane-bounded organelle | 0.00048596 | 2 | 192 | GO:0043232;GO:0043228 | intracellular non-membrane-bounded organelle | Cellular Component |
| 51/121 ribosome | 0.00455291 | 3 | 121 | GO:0005840 | ribosome | Cellular Component |
| 10/11 eukaryotic translation initiation factor 3 complex | 0.00058929 | 2 | 11 | GO:0005852 | eukaryotic translation initiation factor 3 complex | Cellular Component |
| 6/7 signal recognition particle | 0.02206575 | 2 | 7 | GO:0048500 | signal recognition particle | Cellular Component |
| 13/26 ribosomal subunit | 0.08873271 | 2 | 26 | GO:0044391 | ribosomal subunit | Cellular Component |
| 7/11 spliceosomal complex | 0.08991939 | 2 | 11 | GO:0005681 | spliceosomal complex | Cellular Component |
| 96/190 ribonucleoprotein complex | 1.14E-10 | 2 | 7 | GO:0030532;GO:0120114;GO:0097525 | ribonucleoprotein complex | Cellular Component |
| 7/8 COP9 signalosome | 0.01038875 | 2 | 8 | GO:0008180 | COP9 signalosome | Cellular Component |
| 5/5 integrator complex | 0.0172184 | 4 | 5 | GO:0032039 | integrator complex | Cellular Component |
| 10/15 protein targeting | 0.01116985 | 3 | 15 | GO:0006605 | protein targeting | Biological Process |
| 8/9 protein targeting to membrane | 0.00185957 | 5 | 9 | GO:0006614;GO:0006613;GO:0045047;GO:0006612;GO:0072599;GO:0070972 | protein targeting to membrane | Biological Process |
| 17/36 cellular protein localization | 0.03586536 | 2 | 36 | GO:0072594;GO:0033365;GO:0034613;GO:0070727 | cellular protein localization | Biological Process |
| 10/14 protein localization to membrane | 0.00540483 | 3 | 14 | GO:0090150;GO:0072657 | protein localization to membrane | Biological Process |
| 5/7 vacuole organization | 0.09899767 | 2 | 7 | GO:0007033 | vacuole organization | Biological Process |
| 35/71 regulation of protein metabolic process | 0.00017951 | 2 | 71 | GO:0032268;GO:0051246 | regulation of protein metabolic process | Biological Process |
| 26/44 posttranscriptional regulation of gene expression | 4.42E-05 | 2 | 44 | GO:0006417;GO:0010608;GO:0034248 | posttranscriptional regulation of gene expression | Biological Process |
| 21/27 regulation of translational initiation | 5.83E-07 | 5 | 27 | GO:0006446 | regulation of translational initiation | Biological Process |
| 7/10 nucleic acid phosphodiester bond hydrolysis | 0.03621611 | 2 | 10 | GO:0090501;GO:0090305 | nucleic acid phosphodiester bond hydrolysis | Biological Process |
| 20/43 methylation | 0.02270902 | 3 | 43 | GO:0043414;GO:0032259 | methylation | Biological Process |
| 16/37 RNA splicing | 0.09864806 | 2 | 37 | GO:0008380;GO:0000398;GO:0000377;GO:0000375 | RNA splicing | Biological Process |
| 16/23 RNA catabolic process | 0.0001842 | 3 | 23 | GO:0006401 | RNA catabolic process | Biological Process |
| 11/17 mRNA catabolic process | 0.00904014 | 4 | 17 | GO:0006402;GO:0000956 | mRNA catabolic process | Biological Process |
| 36/73 mRNA metabolic process | 0.00013975 | 6 | 73 | GO:0016071;GO:0006397 | mRNA metabolic process | Biological Process |
| 5/7 RNA 3'-end processing | 0.09899767 | 7 | 7 | GO:0031123 | RNA 3'-end processing | Biological Process |
| 7/8 pseudouridine synthesis | 0.00582309 | 2 | 8 | GO:0001522 | pseudouridine synthesis | Biological Process |
| 25/33 RNA modification | 1.08E-07 | 2 | 33 | GO:0009451 | RNA modification | Biological Process |
| 17/20 rRNA metabolic process | 1.23E-06 | 7 | 20 | GO:0016072;GO:0006364 | rRNA metabolic process | Biological Process |
| 64/87 ncRNA metabolic process | 1.00E-15 | 2 | 87 | GO:0034660 | ncRNA metabolic process | Biological Process |
| 43/60 tRNA metabolic process | 3.89E-12 | 3 | 60 | GO:0006399 | tRNA metabolic process | Biological Process |
| 10/13 tRNA modification | 0.00212699 | 2 | 13 | GO:0006400 | tRNA modification | Biological Process |
| 39/53 ncRNA processing | 1.24E-11 | 6 | 53 | GO:0034470 | ncRNA processing | Biological Process |
| 20/29 tRNA processing | 2.46E-05 | 7 | 29 | GO:0008033 | tRNA processing | Biological Process |
| 88/149 RNA processing | 1.05E-15 | 7 | 29 | GO:0008033 | RNA processing | Biological Process |
| 170/273 RNA metabolic process | 1.00E-15 | 2 | 273 | GO:0016070 | RNA metabolic process | Biological Process |
| 7/11 tetrapyrrole metabolic process | 0.06910884 | 2 | 11 | GO:0006778;GO:0033013 | tetrapyrrole metabolic process | Biological Process |
| 5/7 chlorophyll metabolic process | 0.09899767 | 3 | 7 | GO:0015994 | chlorophyll metabolic process | Biological Process |
| 29/68 nucleobase metabolic process | 0.01401744 | 2 | 68 | GO:0006144;GO:0009112 | nucleobase metabolic process | Biological Process |
| 21/34 pyrimidine-containing compound metabolic process | 0.00013975 | 2 | 34 | GO:0006206;GO:0072527 | pyrimidine-containing compound metabolic process | Biological Process |
| 37/60 RNA biosynthetic process | 1.26E-07 | 4 | 60 | GO:0032774 | RNA biosynthetic process | Biological Process |
| 27/38 transcription, DNA-templated | 1.74E-07 | 3 | 38 | GO:0006351;GO:0097659 | transcription, DNA-templated | Biological Process |
| 80/178 organic cyclic compound biosynthetic process | 1.74E-07 | 2 | 178 | GO:0018130;GO:0019438;GO:1901362;GO:0034654 | organic cyclic compound biosynthetic process | Biological Process |
| 86/211 macromolecule biosynthetic process | 7.13E-06 | 4 | 211 | GO:0034645;GO:0009059 | macromolecule biosynthetic process | Biological Process |
| 6/9 transcription initiation from RNA polymerase II promoter | 0.08491833 | 3 | 9 | GO:0006367 | transcription initiation from RNA polymerase II promoter | Biological Process |
| 10/17 DNA-templated transcription, initiation | 0.03563066 | 2 | 17 | GO:0006352 | DNA-templated transcription, initiation | Biological Process |
| 108/277 cellular nitrogen compound biosynthetic process | 3.12E-06 | 2 | 277 | GO:0044271 | cellular nitrogen compound biosynthetic process | Biological Process |
| 13/25 organic hydroxy compound metabolic process | 0.03621611 | 2 | 25 | GO:1901615 | organic hydroxy compound metabolic process | Biological Process |
| 13/19 glycine metabolic process | 0.00150825 | 2 | 19 | GO:0006544;GO:0006563;GO:0006566 | glycine metabolic process | Biological Process |
| 33/83 serine family amino acid metabolic process | 0.02567161 | 2 | 83 | GO:0009069 | serine family amino acid metabolic process | Biological Process |
| 5/6 serine family amino acid biosynthetic process | 0.04549355 | 3 | 6 | GO:0009070 | serine family amino acid biosynthetic process | Biological Process |
| 13/26 regulation of neurotransmitter levels | 0.04982831 | 2 | 26 | GO:0042133;GO:0001505 | regulation of neurotransmitter levels | Biological Process |
| 23/54 aspartate family amino acid metabolic process | 0.03621611 | 2 | 54 | GO:0009066 | aspartate family amino acid metabolic process | Biological Process |
| 23/31 tRNA aminoacylation | 5.83E-07 | 6 | 31 | GO:0006418;GO:0043039;GO:0043038 | tRNA aminoacylation | Biological Process |
| 97/308 oxoacid metabolic process | 0.04982831 | 2 | 308 | GO:0006520;GO:0019752;GO:0043436;GO:0006082;GO:1901605 | oxoacid metabolic process | Biological Process |
| 13/27 glutamine family amino acid metabolic process | 0.06912471 | 5 | 27 | GO:0009064 | glutamine family amino acid metabolic process | Biological Process |
| 7/10 glutamine family amino acid biosynthetic process | 0.03621611 | 3 | 10 | GO:0009084 | glutamine family amino acid biosynthetic process | Biological Process |
| 31/71 carboxylic acid biosynthetic process | 0.00670303 | 2 | 71 | GO:0046394;GO:0016053 | carboxylic acid biosynthetic process | Biological Process |
| 39/108 small molecule biosynthetic process | 0.06181084 | 3 | 108 | GO:0044283 | small molecule biosynthetic process | Biological Process |
| 21/38 cellular amino acid biosynthetic process | 0.0012593 | 4 | 38 | GO:1901607;GO:0008652 | cellular amino acid biosynthetic process | Biological Process |
| 6/8 pteridine-containing compound metabolic process | 0.0424143 | 2 | 8 | GO:0006760;GO:0042558 | pteridine-containing compound metabolic process | Biological Process |
| 58/141 ribonucleoprotein complex biogenesis | 0.00032251 | 3 | 141 | GO:0006412;GO:0043043;GO:0043604;GO:0042254;GO:0022613;GO:0044085 | ribonucleoprotein complex biogenesis | Biological Process |
| 6/9 water-soluble vitamin biosynthetic process | 0.08491833 | 4 | 9 | GO:0042364;GO:0009110 | water-soluble vitamin biosynthetic process | Biological Process |
| 83/229 organonitrogen compound biosynthetic process | 0.00144004 | 2 | 229 | GO:1901566 | organonitrogen compound biosynthetic process | Biological Process |
| 32/80 protein-containing complex subunit organization | 0.02594028 | 2 | 80 | GO:0065003;GO:0043933;GO:0034622 | protein-containing complex subunit organization | Biological Process |
| 6/6 spliceosomal snRNP assembly | 0.00395798 | 2 | 6 | GO:0000387 | spliceosomal snRNP assembly | Biological Process |
| 16/21 ribonucleoprotein complex subunit organization | 3.67E-05 | 4 | 21 | GO:0022618;GO:0071826 | ribonucleoprotein complex subunit organization | Biological Process |
| 25/42 protein folding | 5.25E-05 | 2 | 42 | GO:0006457 | protein folding | Biological Process |
| 9/17 peptidyl-proline modification | 0.09899767 | 4 | 17 | GO:0000413;GO:0018208 | peptidyl-proline modification | Biological Process |
| 22/49 peptidyl-amino acid modification | 0.02354805 | 2 | 49 | GO:0018193 | peptidyl-amino acid modification | Biological Process |
| 26/59 chromatin organization | 0.01401744 | 3 | 59 | GO:0006325 | chromatin organization | Biological Process |
| 9/17 chromatin remodeling | 0.09899767 | 2 | 17 | GO:0006338 | chromatin remodeling | Biological Process |
| 9/13 proteasomal protein catabolic process | 0.01350689 | 3 | 13 | GO:0043161;GO:0010498 | proteasomal protein catabolic process | Biological Process |
| 57/130 DNA metabolic process | 4.62E-05 | 2 | 130 | GO:0006259 | DNA metabolic process | Biological Process |
| 46/90 cellular response to stimulus | 2.97E-06 | 2 | 90 | GO:0006281;GO:0006974;GO:0033554;GO:0051716 | cellular response to stimulus | Biological Process |
| 52/153 response to stimulus | 0.06912471 | 2 | 153 | GO:0006950;GO:0050896 | response to stimulus | Biological Process |
| 0/24 actin filament binding | 0.03985367 | 3 | 24 | GO:0051015 | actin filament binding | Molecular Function |
| 1/55 actin binding | 0.00206534 | 2 | 55 | GO:0003779 | actin binding | Molecular Function |
| 5/119 cytoskeletal protein binding | 1.42E-05 | 3 | 119 | GO:0008092 | cytoskeletal protein binding | Molecular Function |
| 4/54 tubulin binding | 0.03282394 | 5 | 54 | GO:0008017;GO:0015631 | tubulin binding | Molecular Function |
| 1/67 motor activity | 0.00042293 | 7 | 67 | GO:0003774 | motor activity | Molecular Function |
| 1/47 microtubule motor activity | 0.00565243 | 2 | 47 | GO:0003777 | microtubule motor activity | Molecular Function |
| 2/39 signaling receptor binding | 0.04012882 | 3 | 39 | GO:0005102 | signaling receptor binding | Molecular Function |
| 4/5 chaperone binding | 0.01979594 | 2 | 5 | GO:0051087 | chaperone binding | Molecular Function |
| 2/32 enzyme inhibitor activity | 0.09317135 | 3 | 32 | GO:0004857 | enzyme inhibitor activity | Molecular Function |
| 23/153 molecular function regulator | 0.06187415 | 1 | 153 | GO:0098772 | molecular function regulator | Molecular Function |
| 2/47 guanyl-nucleotide exchange factor activity | 0.01603763 | 3 | 47 | GO:0005085 | guanyl-nucleotide exchange factor activity | Molecular Function |
| 9/97 enzyme binding | 0.00884591 | 2 | 97 | GO:0051020;GO:0019899 | enzyme binding | Molecular Function |
| 0/31 Rho GTPase binding | 0.01714944 | 7 | 31 | GO:0017048 | Rho GTPase binding | Molecular Function |
| 0/26 Ras guanyl-nucleotide exchange factor activity | 0.03009607 | 3 | 26 | GO:0005089;GO:0005088 | Ras guanyl-nucleotide exchange factor activity | Molecular Function |
| 4/55 small GTPase binding | 0.02930266 | 5 | 55 | GO:0017016;GO:0031267 | small GTPase binding | Molecular Function |
| 1/31 protein tyrosine kinase activity | 0.04470199 | 3 | 31 | GO:0004713 | protein tyrosine kinase activity | Molecular Function |
| 14/144 molecular transducer activity | 0.0015028 | 2 | 144 | GO:0004888;GO:0038023;GO:0060089 | molecular transducer activity | Molecular Function |
| 3/78 G protein-coupled receptor activity | 0.00077363 | 2 | 78 | GO:0004930 | G protein-coupled receptor activity | Molecular Function |
| 1/30 phosphatidylinositol binding | 0.04787038 | 2 | 30 | GO:0035091 | phosphatidylinositol binding | Molecular Function |
| 2/45 phospholipid binding | 0.01979594 | 2 | 45 | GO:0005543 | phospholipid binding | Molecular Function |
| 5/66 lipid binding | 0.01794637 | 2 | 66 | GO:0008289 | lipid binding | Molecular Function |
| 15/222 calcium ion binding | 6.97E-08 | 5 | 222 | GO:0005509 | calcium ion binding | Molecular Function |
| 13/24 unfolded protein binding | 0.00415066 | 3 | 24 | GO:0051082 | unfolded protein binding | Molecular Function |
| 5/50 tetrapyrrole binding | 0.0593206 | 2 | 50 | GO:0020037;GO:0046906 | tetrapyrrole binding | Molecular Function |
| 6/10 4 iron, 4 sulfur cluster binding | 0.03695008 | 2 | 10 | GO:0051539 | 4 iron, 4 sulfur cluster binding | Molecular Function |
| 5/8 histone acetyltransferase activity | 0.0468365 | 9 | 8 | GO:0004402;GO:0061733;GO:0034212 | histone acetyltransferase activity | Molecular Function |
| 38/108 structural constituent of ribosome | 0.02193128 | 2 | 108 | GO:0003735 | structural constituent of ribosome | Molecular Function |
| 20/134 ion transmembrane transporter activity | 0.08443052 | 2 | 134 | GO:0015318;GO:0015075 | ion transmembrane transporter activity | Molecular Function |
| 4/50 monovalent inorganic cation transmembrane transporter activity | 0.04974107 | 4 | 50 | GO:0015077 | monovalent inorganic cation transmembrane transporter activity | Molecular Function |
| 1/25 proton transmembrane transporter activity | 0.09317135 | 5 | 25 | GO:0015078 | proton transmembrane transporter activity | Molecular Function |
| 5/6 proteasome-activating ATPase activity | 0.00552191 | 2 | 6 | GO:0036402 | proteasome-activating ATPase activity | Molecular Function |
| 23/33 helicase activity | 1.26E-08 | 2 | 33 | GO:0004386 | helicase activity | Molecular Function |
| 8/13 DNA helicase activity | 0.01023059 | 6 | 13 | GO:0003678 | DNA helicase activity | Molecular Function |
| 6/9 ATP-dependent DNA helicase activity | 0.01724068 | 2 | 9 | GO:0004003 | ATP-dependent DNA helicase activity | Molecular Function |
| 9/14 DNA-dependent ATPase activity | 0.00360833 | 2 | 14 | GO:0008094 | DNA-dependent ATPase activity | Molecular Function |
| 33/91 ATPase activity | 0.02193128 | 7 | 91 | GO:0016887 | ATPase activity | Molecular Function |
| 9/13 purine NTP-dependent helicase activity | 0.0015028 | 7 | 91 | GO:0016887 | purine NTP-dependent helicase activity | Molecular Function |
| 26/64 ATPase activity, coupled | 0.00927534 | 2 | 64 | GO:0042623 | ATPase activity, coupled | Molecular Function |
| 16/117 DNA-binding transcription factor activity | 0.05915589 | 2 | 117 | GO:0003700 | DNA-binding transcription factor activity | Molecular Function |
| 85/293 DNA binding | 0.07712914 | 3 | 293 | GO:0003677 | DNA binding | Molecular Function |
| 5/9 damaged DNA binding | 0.09362255 | 2 | 9 | GO:0003684 | damaged DNA binding | Molecular Function |
| 15/19 5'-3' RNA polymerase activity | 3.45E-07 | 5 | 19 | GO:0003899;GO:0034062;GO:0097747 | 5'-3' RNA polymerase activity | Molecular Function |
| 25/49 nucleotidyltransferase activity | 1.00E-04 | 2 | 49 | GO:0016779 | nucleotidyltransferase activity | Molecular Function |
| 10/16 peptidyl-prolyl cis-trans isomerase activity | 0.00284725 | 2 | 16 | GO:0003755;GO:0016859 | peptidyl-prolyl cis-trans isomerase activity | Molecular Function |
| 7/13 intramolecular transferase activity | 0.04787038 | 2 | 13 | GO:0016866 | intramolecular transferase activity | Molecular Function |
| 5/6 pseudouridine synthase activity | 0.00552191 | 3 | 6 | GO:0009982 | pseudouridine synthase activity | Molecular Function |
| 23/54 isomerase activity | 0.00764641 | 2 | 54 | GO:0016853 | isomerase activity | Molecular Function |
| 4/6 carboxyl-O-methyltransferase activity | 0.05915589 | 4 | 6 | GO:0051998;GO:0010340 | carboxyl-O-methyltransferase activity | Molecular Function |
| 13/31 S-adenosylmethionine-dependent methyltransferase activity | 0.06545627 | 3 | 31 | GO:0008757 | S-adenosylmethionine-dependent methyltransferase activity | Molecular Function |
| 33/64 transferase activity, transferring one-carbon groups | 2.42E-06 | 2 | 64 | GO:0008168;GO:0016741 | transferase activity, transferring one-carbon groups | Molecular Function |
| 8/11 RNA methyltransferase activity | 0.001598 | 3 | 11 | GO:0008173 | RNA methyltransferase activity | Molecular Function |
| 5/8 oxidoreductase activity, acting on the CH-NH group of donors | 0.0468365 | 2 | 8 | GO:0016646;GO:0016645 | oxidoreductase activity, acting on the CH-NH group of donors | Molecular Function |
| 11/15 ligase activity, forming carbon-nitrogen bonds | 0.00010458 | 2 | 15 | GO:0016879 | ligase activity, forming carbon-nitrogen bonds | Molecular Function |
| 4/5 ubiquitin-like modifier activating enzyme activity | 0.01979594 | 4 | 5 | GO:0008641 | ubiquitin-like modifier activating enzyme activity | Molecular Function |
| 5/8 ribonucleoprotein complex binding | 0.0468365 | 2 | 8 | GO:0043021 | ribonucleoprotein complex binding | Molecular Function |
| 19/51 nuclease activity | 0.0817132 | 4 | 51 | GO:0004518 | nuclease activity | Molecular Function |
| 7/12 endoribonuclease activity | 0.02717496 | 2 | 12 | GO:0004521 | endoribonuclease activity | Molecular Function |
| 9/20 endonuclease activity | 0.09317135 | 5 | 20 | GO:0004519 | endonuclease activity | Molecular Function |
| 6/7 endoribonuclease activity, producing 5'-phosphomonoesters | 0.0015028 | 3 | 7 | GO:0016891;GO:0016893 | endoribonuclease activity, producing 5'-phosphomonoesters | Molecular Function |
| 4/5 RNA helicase activity | 0.01979594 | 2 | 5 | GO:0003724 | RNA helicase activity | Molecular Function |
| 64/101 catalytic activity, acting on RNA | 1.00E-15 | 2 | 101 | GO:0140098 | catalytic activity, acting on RNA | Molecular Function |
| 32/44 catalytic activity, acting on a tRNA | 8.17E-13 | 3 | 44 | GO:0140101 | catalytic activity, acting on a tRNA | Molecular Function |
| 23/31 ligase activity, forming carbon-oxygen bonds | 1.06E-09 | 3 | 31 | GO:0004812;GO:0016875 | ligase activity, forming carbon-oxygen bonds | Molecular Function |
| 39/61 ligase activity | 3.72E-12 | 2 | 61 | GO:0016874 | ligase activity | Molecular Function |
| 20/25 translation initiation factor activity | 1.06E-09 | 2 | 25 | GO:0003743 | translation initiation factor activity | Molecular Function |
| 21/34 translation regulator activity | 3.12E-06 | 4 | 34 | GO:0008135;GO:0090079;GO:0045182 | translation regulator activity | Molecular Function |
| 5/6 7S RNA binding | 0.00552191 | 2 | 6 | GO:0008312 | 7S RNA binding | Molecular Function |
| 6/7 tRNA binding | 0.0015028 | 2 | 7 | GO:0000049 | tRNA binding | Molecular Function |
| 98/206 RNA binding | 5.86E-15 | 2 | 206 | GO:0003723 | RNA binding | Molecular Function |
| 9/14 threonine-type peptidase activity | 0.00360833 | 5 | 14 | GO:0004298;GO:0070003 | threonine-type peptidase activity | Molecular Function |
| 9/16 carbon-carbon lyase activity | 0.01603763 | 3 | 16 | GO:0016830;GO:0016831 | carbon-carbon lyase activity | Molecular Function |

Supplementary table 7: Brown module

| **Brown Module** |  |  |  |  |  |  |  |
| --- | --- | --- | --- | --- | --- | --- | --- |
| **Composition** | **pval** | **level** | **nseqs** | **term** | **name** | **GO_Category** | |
| 6/8 collagen-containing extracellular matrix | 0.00079211 | 3 | 8 | GO:0062023 | collagen-containing extracellular matrix | Cellular Component | |
| 13/17 extracellular matrix | 8.32E-09 | 2 | 17 | GO:0031012 | extracellular matrix | Cellular Component | |
| 17/27 extracellular region part | 2.51E-09 | 1 | 27 | GO:0044421 | extracellular region part | Cellular Component | |
| 14/55 plasma membrane part | 0.01976006 | 2 | 55 | GO:0044459 | plasma membrane part | Cellular Component | |
| 10/39 plasma membrane protein complex | 0.07877059 | 2 | 39 | GO:0098797 | plasma membrane protein complex | Cellular Component | |
| 8/26 extracellular region | 0.06701232 | 1 | 26 | GO:0005576 | extracellular region | Cellular Component | |
| 11/22 plasma membrane | 9.80E-05 | 2 | 22 | GO:0005886 | plasma membrane | Cellular Component | |
| 23/56 cell adhesion | 5.40E-05 | 2 | 56 | GO:0007155;GO:0022610 | cell adhesion | Biological Process | |
| 12/26 cell-cell adhesion | 0.00800836 | 5 | 26 | GO:0007156;GO:0098742;GO:0098609 | cell-cell adhesion | Biological Process | |
| 26/93 G protein-coupled receptor signaling pathway | 0.01155624 | 4 | 93 | GO:0007186 | G protein-coupled receptor signaling pathway | Biological Process | |
| 8/17 multicellular organismal process | 0.07465798 | 1 | 17 | GO:0032501 | multicellular organismal process | Biological Process | |
| 14/40 oxidoreductase activity, acting on paired donors, with incorporation or reduction of molecular oxygen | 0.06007959 | 2 | 40 | GO:0016705 | oxidoreductase activity, acting on paired donors, with incorporation or reduction of molecular oxygen | Molecular Function | |
| 36/144 molecular transducer activity | 0.06007959 | 3 | 144 | GO:0004888;GO:0038023;GO:0060089 | molecular transducer activity | Molecular Function | |
| 23/78 G protein-coupled receptor activity | 0.05856424 | 2 | 78 | GO:0004930 | G protein-coupled receptor activity | Molecular Function | |
| 6/10 cargo receptor activity | 0.06007959 | 2 | 10 | GO:0005044;GO:0038024 | cargo receptor activity | Molecular Function | |
| 22/66 lipid binding | 0.01466812 | 2 | 66 | GO:0008289 | lipid binding | Molecular Function | |
| 9/9 extracellular matrix structural constituent | 5.72E-06 | 2 | 9 | GO:0005201 | extracellular matrix structural constituent | Molecular Function | |
| 65/222 calcium ion binding | 5.16E-06 | 2 | 222 | GO:0005509 | calcium ion binding | Molecular Function | |

Supplementary table 8: Grey60 module

| **Composition** | **pval** | **level** | **nseqs** | **term** | **name** | **GO_Category** |
| --- | --- | --- | --- | --- | --- | --- |
| 3/16 inorganic anion transport | 0.07076842 | 2 | 16 | GO:0015698 | inorganic anion transport | Biological Process |
| 2/5 sulfur compound transport | 0.07797924 | 2 | 5 | GO:0072348 | sulfur compound transport | Biological Process |
| 10/137 drug metabolic process | 0.00351921 | 2 | 137 | GO:0017144 | drug metabolic process | Biological Process |
| 5/23 tricarboxylic acid cycle | 0.00351921 | 6 | 23 | GO:0006099;GO:0006101;GO:0072350 | tricarboxylic acid cycle | Biological Process |
| 6/39 antibiotic metabolic process | 0.00351921 | 2 | 39 | GO:0016999 | antibiotic metabolic process | Biological Process |
| 8/106 purine-containing compound metabolic process | 0.01190916 | 2 | 106 | GO:0072521 | purine-containing compound metabolic process | Biological Process |
| 6/76 ribose phosphate metabolic process | 0.05106864 | 2 | 76 | GO:0019693;GO:0009150;GO:0006163;GO:0009259 | ribose phosphate metabolic process | Biological Process |
| 7/108 nucleoside phosphate metabolic process | 0.05542061 | 4 | 108 | GO:0009117;GO:0006753 | nucleoside phosphate metabolic process | Biological Process |
| 9/156 nucleobase-containing small molecule metabolic process | 0.03055338 | 2 | 156 | GO:0055086 | nucleobase-containing small molecule metabolic process | Biological Process |
| 7/120 cofactor metabolic process | 0.07797924 | 3 | 120 | GO:0051186 | cofactor metabolic process | Biological Process |
| 5/58 monocarboxylic acid metabolic process | 0.07076842 | 3 | 58 | GO:0032787 | monocarboxylic acid metabolic process | Biological Process |
| 7/45 monosaccharide metabolic process | 0.00189768 | 2 | 45 | GO:0019318;GO:0005996 | monosaccharide metabolic process | Biological Process |
| 9/111 carbohydrate metabolic process | 0.00351921 | 2 | 111 | GO:0005975 | carbohydrate metabolic process | Biological Process |
| 5/32 carbohydrate biosynthetic process | 0.00841752 | 2 | 32 | GO:0006094;GO:0006006;GO:0019319;GO:0046364;GO:0016051 | carbohydrate biosynthetic process | Biological Process |
| 5/26 hydrolase activity, acting on carbon-nitrogen (but not peptide) bonds | 0.00663331 | 4 | 15 | GO:0016811 | hydrolase activity, acting on carbon-nitrogen (but not peptide) bonds, in linear amides | Molecular Function |
| 3/16 carbon-carbon lyase activity | 0.05972791 | 3 | 16 | GO:0016830;GO:0016831 | carbon-carbon lyase activity | Molecular Function |
| 8/109 coenzyme binding | 0.01004091 | 3 | 109 | GO:0050662 | coenzyme binding | Molecular Function |
| 4/36 vitamin binding | 0.05972791 | 2 | 36 | GO:0019842 | vitamin binding | Molecular Function |
| 10/182 cofactor binding | 0.01102748 | 2 | 182 | GO:0048037 | cofactor binding | Molecular Function |
| 4/32 enzyme inhibitor activity | 0.05317122 | 3 | 32 | GO:0004857 | enzyme inhibitor activity | Molecular Function |
| 3/6 cysteine-type endopeptidase inhibitor activity | 0.00733608 | 3 | 6 | GO:0004869 | cysteine-type endopeptidase inhibitor activity | Molecular Function |
| 3/21 anion transmembrane transporter activity | 0.09362351 | 3 | 21 | GO:0015103;GO:0008509 | anion transmembrane transporter activity | Molecular Function |
| 2/5 sulfur compound transmembrane transporter activity | 0.06911822 | 2 | 5 | GO:1901682 | sulfur compound transmembrane transporter activity | Molecular Function |

Blue module:

| **Composition** | **pval** | **level** | **nseqs** | **term** | **name** | **GO_Category** |
| --- | --- | --- | --- | --- | --- | --- |
| 5/7 synapse part | 0.07020436 | 1 | 7 | GO:0044456 | synapse part | Cellular Component |
| 5/7 integral component of endoplasmic reticulum membrane | 0.07020436 | 4 | 7 | GO:0030176;GO:0031227 | integral component of endoplasmic reticulum membrane | Cellular Component |
| 11/21 myosin complex | 0.02747024 | 2 | 21 | GO:0016459 | myosin complex | Cellular Component |
| 4/5 dynactin complex | 0.07170889 | 5 | 5 | GO:0005869 | dynactin complex | Cellular Component |
| 22/64 supramolecular fiber | 0.07020436 | 3 | 64 | GO:0005874;GO:0099513;GO:0099512;GO:0099081;GO:0099080 | supramolecular fiber | Cellular Component |
| 19/54 tubulin complex | 0.07020436 | 2 | 54 | GO:0045298 | tubulin complex | Cellular Component |
| 8/16 centrosome | 0.07170889 | 2 | 16 | GO:0005813 | centrosome | Cellular Component |
| 54/154 cytoskeletal part | 0.00024285 | 2 | 154 | GO:0044430 | cytoskeletal part | Cellular Component |
| 21/44 mitochondrial membrane | 0.00140828 | 2 | 44 | GO:0005741;GO:0031966;GO:0031968;GO:0019867;GO:0098805 | mitochondrial membrane | Cellular Component |
| 23/55 organelle membrane | 0.00517492 | 2 | 55 | GO:0031090;GO:0098588 | organelle membrane | Cellular Component |
| 12/26 extracellular region | 0.05295557 | 1 | 26 | GO:0005576 | extracellular region | Cellular Component |
| 46/165 transcription factor complex | 0.07020436 | 2 | 165 | GO:0005667 | transcription factor complex | Cellular Component |
| 10/15 secretion | 0.03717901 | 2 | 15 | GO:0032940;GO:0046903 | secretion | Biological Process |
| 23/49 protein catabolic process | 0.02966291 | 3 | 49 | GO:0030163 | protein catabolic process | Biological Process |
| 20/27 cellular protein catabolic process | 7.42E-06 | 3 | 27 | GO:0035694;GO:0044257 | cellular protein catabolic process | Biological Process |
| 16/34 immune system process | 0.06217081 | 1 | 34 | GO:0002376 | immune system process | Biological Process |
| 22/50 regulation of apoptotic process | 0.04338217 | 6 | 50 | GO:0042981;GO:0043067;GO:0010941 | regulation of apoptotic process | Biological Process |
| 22/52 cytoskeleton organization | 0.06217081 | 2 | 52 | GO:0007010 | cytoskeleton organization | Biological Process |
| 10/15 actin cytoskeleton organization | 0.03717901 | 2 | 15 | GO:0030036;GO:0030029 | actin cytoskeleton organization | Biological Process |
| 29/72 regulation of hydrolase activity | 0.04338217 | 2 | 72 | GO:0043087;GO:0051336 | regulation of hydrolase activity | Biological Process |
| 80/262 phosphorylation | 0.06217081 | 4 | 262 | GO:0016310;GO:0006468 | phosphorylation | Biological Process |
| 44/128 intracellular signal transduction | 0.06217081 | 4 | 128 | GO:0035556 | intracellular signal transduction | Biological Process |
| 6/7 catalase activity | 0.05020166 | 2 | 7 | GO:0004096 | catalase activity | Molecular Function |
| 84/222 calcium ion binding | 0.00093457 | 2 | 222 | GO:0005509 | calcium ion binding | Molecular Function |
| 16/31 Rho GTPase binding | 0.04773316 | 7 | 31 | GO:0017048 | Rho GTPase binding | Molecular Function |
| 29/67 motor activity | 0.02963115 | 2 | 67 | GO:0003774 | motor activity | Molecular Function |
| 15/24 actin filament binding | 0.00598578 | 2 | 24 | GO:0051015 | actin filament binding | Molecular Function |
| 27/55 actin binding | 0.00572628 | 4 | 55 | GO:0003779 | actin binding | Molecular Function |
| 49/119 cytoskeletal protein binding | 0.00473299 | 3 | 119 | GO:0008092 | cytoskeletal protein binding | Molecular Function |

Supplementary Figure 1a: PCAs showing separation based on outplant sites (Grand Isle and LUMCON)


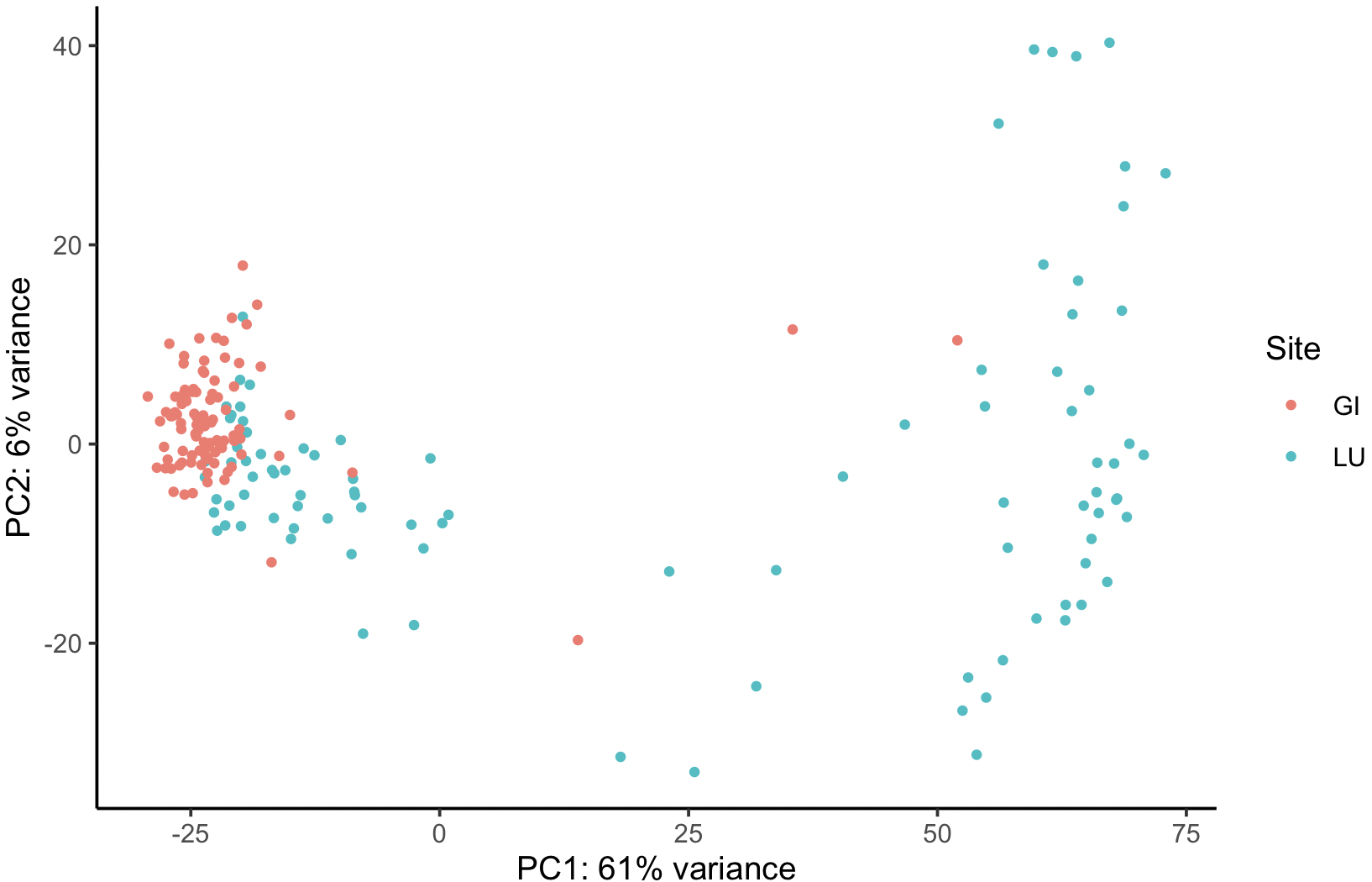


Supplementary Figure 1b: PCAs showing separation based on cohort (LSU (LS) and Auburn (AU))


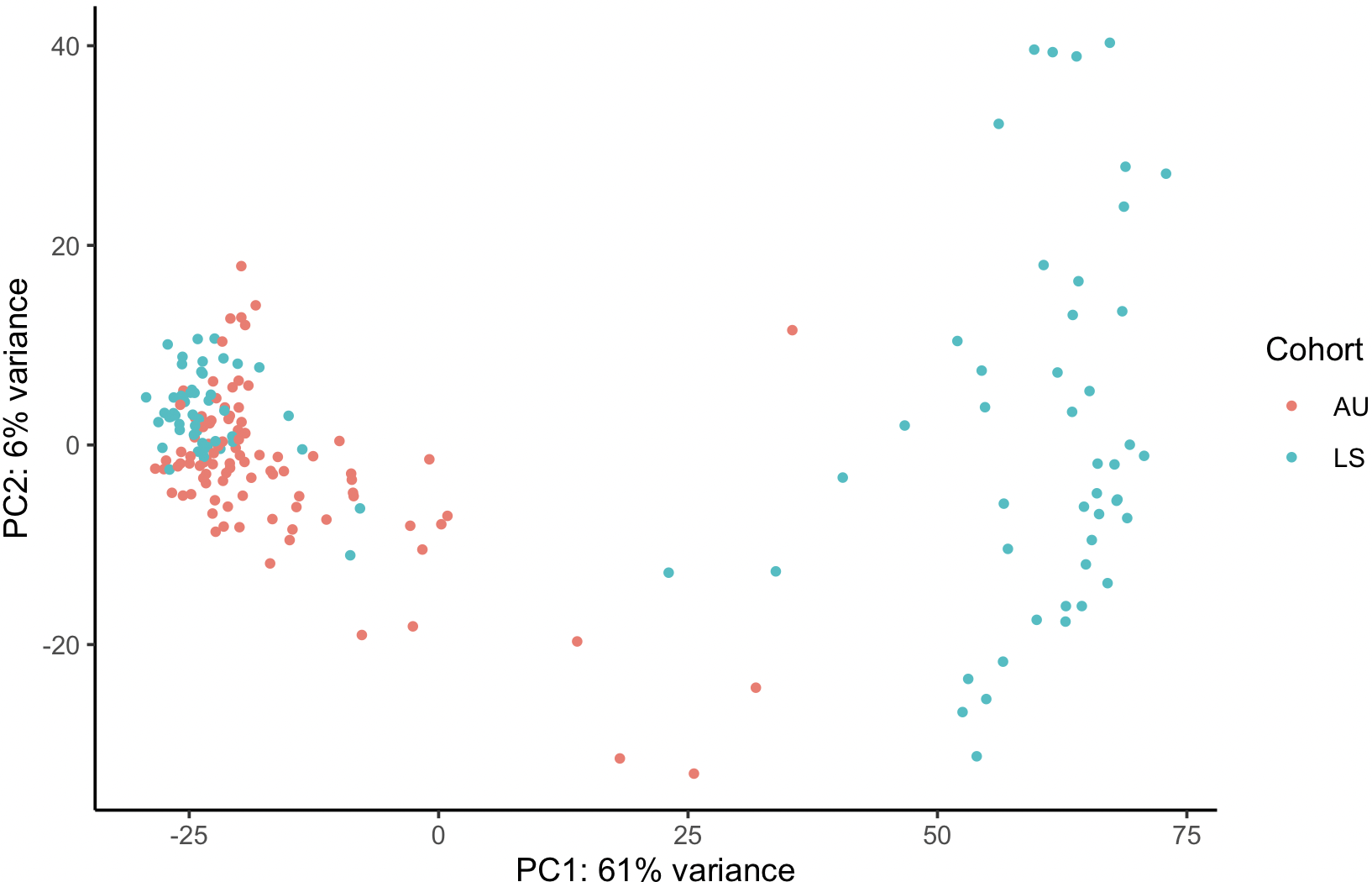


Supplementary Figure 1c: PCAs showing separation based on ploidy (diploid(2n) and triploid(3n))


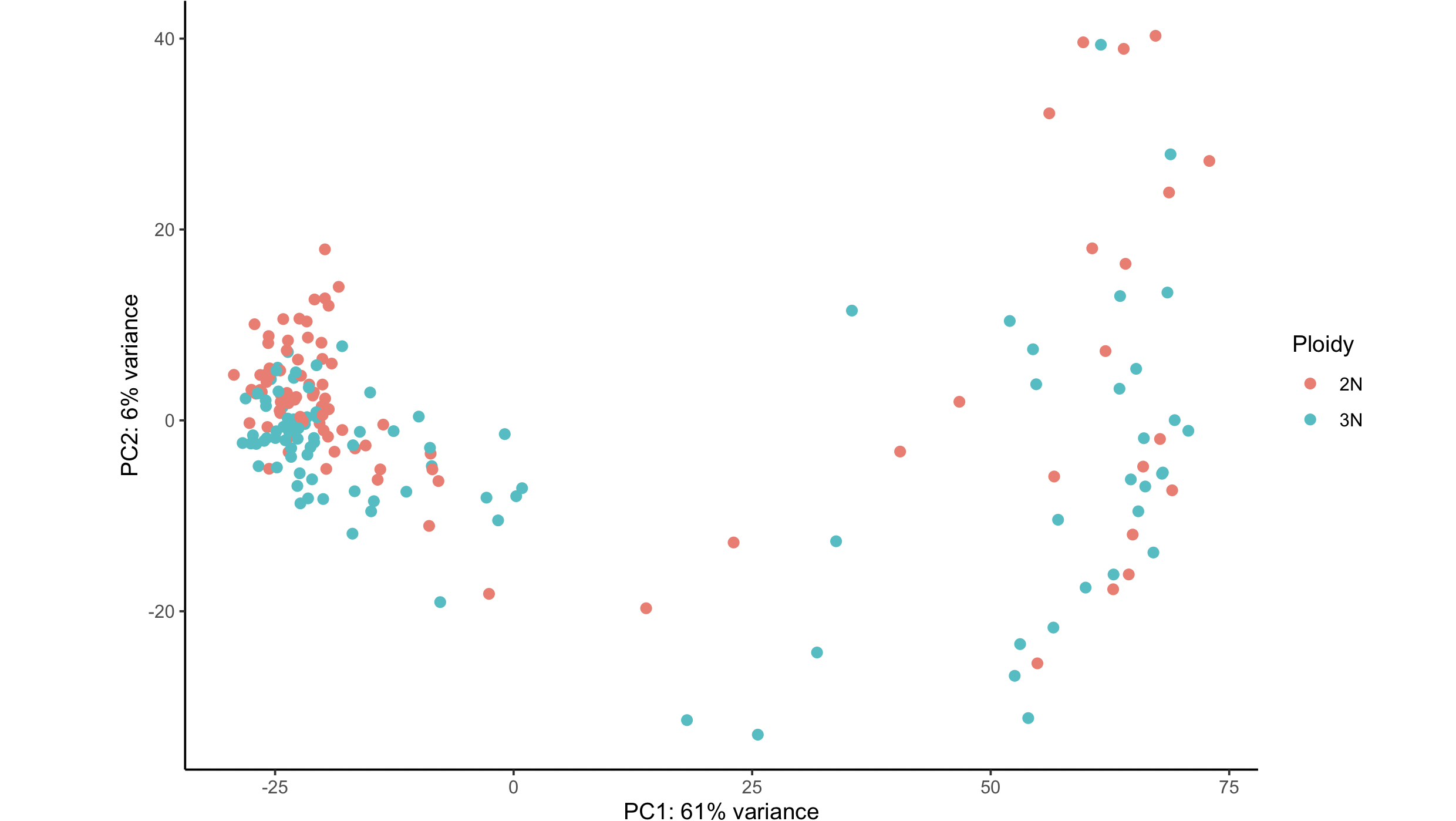


Supplementary Figure 1d: PCAs showing separation based on dams (CL, VB, SL)


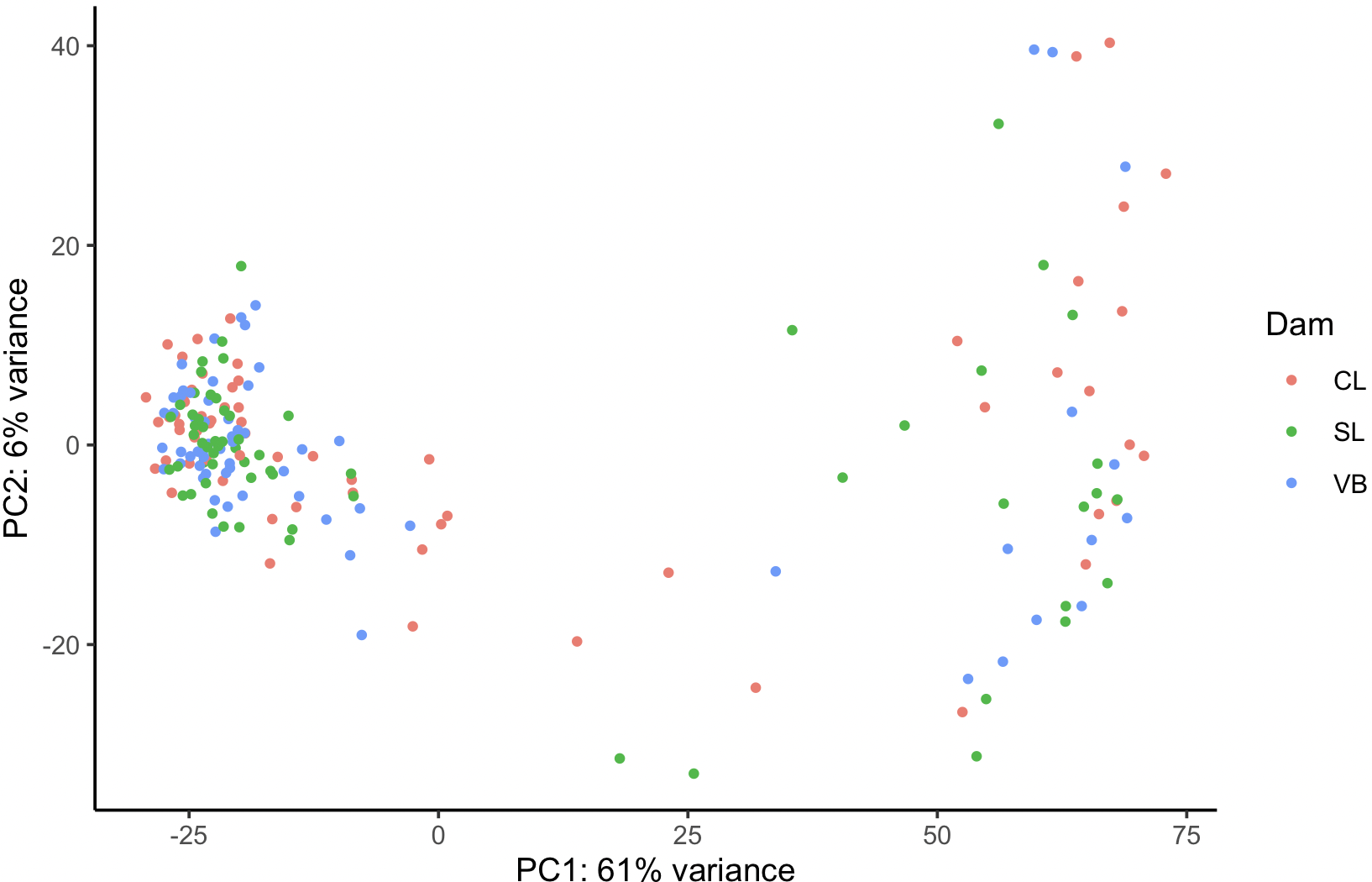

Supplement: Supplementary file 7 — Data S5. [file EVA-17-e70028-s002.docx]
